# Evidence to inform intersectoral policies: a comparison of health and transport sector evidence in support of road traffic injury prevention

**DOI:** 10.1186/s12961-015-0008-9

**Published:** 2015-03-25

**Authors:** James Bao, Kavi Bhalla, Sara Bennett

**Affiliations:** Health Systems Program, Department of International Health, Johns Hopkins Bloomberg School of Public Health, Baltimore, USA

**Keywords:** Health in all policies, Intersectoral action, Research evidence, Road traffic injury prevention

## Abstract

**Background:**

Health is influenced by determinants beyond the traditional conception of the health sector. Increasingly, global actors are targeting policymakers at global and national levels to take an intersectoral approach to health issues. Multilateral organizations in the health and transport communities have published policy reports targeting policymakers to address the burden of road traffic injuries. However, these reports stem from sectors grounded in different disciplinary perspectives. We investigate whether sectors have differing evidentiary traditions by analyzing differences regarding author networks, type of evidence cited, recommendations, and indicators.

**Methods:**

We selected global policy reports on road traffic injury prevention based upon expert opinion and categorized them by sector according to their institutional publisher. For each report, we i) conducted an authorship analysis by sectoral affiliation; ii) analyzed the types of research evidence cited and categorized the evidence type and institutional nature of the publisher; iii) analyzed key recommendations by extracting recommendations presented in the concluding sections of the documents; and iv) examined the use of indicators. Descriptive statistics were used to determine whether dimensions differed by the sectoral affiliation of the policy report.

**Results:**

Authorship was dominated by the sector from which the report was published, while reports that involved both sectors often showed clustering of authors in one sector or another, depending on the subject addressed. Reports originating from different sectors preferentially cited different types of evidence; notably, health sector reports emphasized observational studies and reviews, while transport sector reports drew heavily on government agency reports. There were no differences in recommendations and indicators used.

**Conclusions:**

Notions of knowledge validity and valuations of evidence vary depending on the field’s historical development. Such differences in valuing evidence within sectors may have the potential to undermine the application of evidence in intersectoral policymaking. Strategies to address this challenge include the identification of key individuals to connect separate sectors, knowledge translation activities that take account of sectoral differences, and the tailoring of messages to different audiences. Future analyses on other intersectoral issues may provide clarity on points of tension and differing types of evidence used in intersectoral work.

## Background

The health and well-being of individuals and populations are dependent upon a multitude of complex factors, not strictly confined to the traditional conception of the health sector. Social, economic, and environmental influences play a central role in an individual’s ability to achieve a state of “*complete physical, mental, and social well-being*” ([1], p. 1). Accordingly, governments must address factors beyond the purview of the health sector, and embrace the use of intersectoral approaches including, but not limited to, the finance, agriculture, transport, and social sectors. In contrast to multisectoral or transectoral action, intersectoral action refers to “*actions undertaken by sectors outside the health sector, possibly, but not necessarily, in collaboration with the health sector, on health or health equity outcomes, or on the determinants of health or health equity*” ([2], p. 2), and has been increasingly recognized as a necessary strategy to improve health outcomes and equity. The 2005 World Health Organization (WHO) Commission on the Social Determinants of Health emphasized the role of the social sectors in addressing health inequities [3]; the recent United Nations (UN) General Assembly report on the prevention and control of non-communicable diseases emphasized the need for ‘health in all policies’ approaches [4], and the WHO identified intersectoral action as a key driver and strategy in achieving universal health coverage [5].

Research evidence, that is knowledge produced through a systematic, “*unbiased and objective process of enquiry*” [6] is a key input into the policy process, and interacts with a context’s institutional constraints, interest groups, ideas, and external factors to influence the policy process [7]. Evidence can help frame the problem, clarify what interventions can be used, how they can be delivered, and how to implement the change [8]. The Adelaide Statement for Health in all Policies called for the health sector to strengthen its role in providing an evidence base for intersectoral policies and action, but lacked insight on how health sector views of evidence may (or may not) match with perspectives in other sectors [9]. Literature exploring the nature of the evidence base underlying intersectoral action is extremely limited, but there is suggestion that the technical nature of research evidence may hinder the collaborative uptake of evidence during intersectoral action, and that it is critical to present evidence in a way that non-health sector actors can easily understand and interpret [2]. For the purposes of this paper, we consider research evidence to be information that has been collected and analyzed in a systematic, unbiased, and objective fashion; however, we remain agnostic about the nature of study designs or methodological approaches used, recognizing that different sectors employ differing research strategies.

Difficulties that stakeholders in other sectors face in understanding health research evidence may be due to differing scientific paradigms across sectors. Scientific paradigms are constituted by sets of norms, values, and beliefs that are used to generate, communicate, and validate knowledge. Specialized scientific paradigms form when new models of science displace past competing models in solving relevant problems specific to their field [10]. As findings continue to be communicated, the audience for new discoveries shifts from the general populace to colleagues who are familiar with the rules, theories, and assumptions of the paradigm to which they belong. This allows investigators to ‘start where they left off’ and target their scientific findings to their scientific peers [10]. Consequently, those working outside the shared paradigm are increasingly unable to interpret the evidence. Sectors of government and academic faculty and departments often exist within their own communities, dependent on existing institutional structures [11], and drawing on research evidence from their respective fields to inform decision-making. When working intersectorally, however, research evidence may stem from unfamiliar paradigms and policymakers may encounter difficulty comprehending the evidence from scientific paradigms to which they do not belong. Further, this unfamiliarity with other research paradigms may impact the perceived legitimacy of evidence put forth, and how such evidence is prioritized in informing decision-making.

In public health, some of the most pressing issues must be addressed through intersectoral action. For example, obesity is increasingly addressed intersectorally, with action from the health, education, social, transport, and food sectors [12-16]. Similarly, efforts to reduce the burden of zoonotic diseases harness the expertise of the agriculture, environment, education, biomedical, and health sectors [17-19]. Nutrition policy in both high-income and low- and middle-income contexts has also taken intersectoral approaches, combining public health with agriculture, economics, and food security to tackle nutritional issues [20-24].

In order to enhance understanding about the degree to which alternative evidentiary paradigms might work to inhibit intersectoral action, we investigate the nature of research evidence in documents originating across the health and transport sectors as applied to road traffic injury prevention (RTIP). RTIP is an appropriate case for our analysis given the fact that i) it represents a leading cause of death among adolescents worldwide, accounting for the loss of approximately 1.2 million lives and between 20 to 50 million injuries every year, creating economic burdens of up to 2% of GDP annually [25]. ii) It involves two distinct sectors with, apparently, quite different scientific paradigms. On the health side, RTIP policy has been primarily framed within a traditional public health paradigm that parallels the host-agent-environment model in epidemiology [26]. On the transport side, our anticipation was that RTIP had been primarily framed within an engineering paradigm. However, our research and consultation with a RTIP expert led us to understand a broader set of approaches that have influenced transport sector perspectives, which now increasingly include investigation from both an engineering and social/behavioral science frame. iii) There is growing interest in RTIP; global actors in health and transport are increasingly targeting policymakers through policy reports that act as guides and an evidentiary basis for implementing evidence-informed policy interventions to reduce the burden of road traffic injuries [27-30].

Through an analysis of prominent international reports on RTIP published by major health and transport organizations, we sought to investigate whether there are differences between the health and transport reports with respect to i) author networks; ii) interventions and types of evidence cited; iii) recommendations; and iv) indicators used.

## Methods

We examined the nature of research evidence by selecting influential policy documents on global RTIP and categorizing them based on the mission and orientation of the publishing organization. Through discussions with an expert in the RTIP field (KB) who straddles both the health and engineering communities, we identified the major organizational players in the field and selected one major organization stemming from each of the fields of health, transport, and mixed.

We selected documents from the WHO, World Bank (WB), and the Organization for Economic Cooperation and Development (OECD), as they represent the most prominent organizations operating within the health, transport, and economic fields on an international level. The WHO is the premier international agency responsible for health within the UN system and is responsible for coordinating all RTIP work within it. The WB is an international development bank, with a mandate that encompasses both public health and infrastructure development (including transport); it therefore represents a mixed (health and transport) perspective on RTIP. The WB takes behavior change, capacity building, and systems engineering approaches to address RTIP and its associated economic consequences. The OECD collaborates with the International Transport Forum (ITF) to contribute to international co-operation on road accidents. While the OECD does have a division for health under the Directorate for Employment, Labour and Social Affairs, to date, its work on road traffic injury has been driven largely by the ITF. The ITF was created under a Declaration issued by the Council of Ministers of the European Conference of Ministers of Transport, and thus has a predominantly transport perspective. From these three organizations, we included all reports that were i) aimed at policymakers; ii) included an evidentiary basis utilizing research evidence on RTIP interventions that were included in the landmark 2004 Report on Road Traffic Injury Prevention [25]; iii) formed recommendations; and iv) were published after 2002. We considered reports to be aimed at policymakers if they provided evidence for interventions and outlined implementation considerations.

For each report, we first conducted an authorship analysis to study formations of authorship networks [31,32]. We extracted all listed authors’ sectoral affiliation (health, mixed, or transport) from each document, based upon the organization that they currently work at. Second, we analyzed the types of research evidence cited in each report. We centered this on seven RTIP intervention areas that were prevalent across the selected documents: alcohol use, road design, seatbelts and restraints, vehicle design, speed limits, speeding and helmet use. From each report, and for each intervention area, JB extracted the citations used to support writing on the intervention area, and categorized the evidence type (Table 1) and the institutional nature of the publisher (e.g., peer-review journal, government agency report, etc.). For each citation, the abstract (when available) was used to determine the type of evidence. The full document was used when an abstract was unclear or unavailable. The results were tallied to compare the types of evidence cited, and institutional affiliations included across the health, transport, and mixed reports. Third, we analyzed key recommendations by extracting recommendations presented in the concluding or recommendation sections of the documents. Within these sections, the main points of emphasis, shown by bolding or placement as a subtitle, were extracted as the key recommendations. The purpose of this exercise was to investigate whether different sectors reporting on RTIP drew similar or different conclusions based on the use of different types of evidence. Fourth, we examined indicators employed in the reports to determine whether different indicators were used in different sectors to measure and quantify road traffic injuries, as we hypothesized that the health and transport sectors may measure deaths and crashes with different denominators (population and distance traveled, respectively). We based our analysis on the list of the most commonly used RTIP indicators, as indicated by the WHO 2004 report on Road Traffic Injury Prevention [25]. These indicators were tracked across our 12 selected reports, and we noted whether the indicator was either explicitly mentioned, used, or absent in the documents. We compared whether the use of these indicators differed across reports from different sectors. The lead investigator (JB) was responsible for all data extraction from the reports, which was reviewed by SB, followed by discussion by both authors on the categorization of data extracted.

**Table 1:** **Description of evidence types used to categorize evidence cited**

	**Type of evidence**	**Description**
**Research evidence type**	Case study	Document detailing the experience of a particular road traffic injury prevention initiative, with or without analysis and recommendations from the experience
	Economic analyses	Document using economic methods to model finances associated with an intervention
	Experimental	Experimental study with assigned interventions
		Includes randomized controlled trials, quasi-experimental trials
	Guidelines	Document detailing best practices or defining best practices for the implementation of road traffic injury prevention programs/interventions
	Mechanical simulation test	Document detailing the results of mechanical simulation tests
		Includes tests of vehicle design, and physical interventions
	Modeling	Study using statistical and/or computerized models to predict the outcomes and impacts of implementing interventions
	Observational	Research articles using study designs including retrospective cohort, prospective cohort, before/after, case control and time-series designs
	Other	Regulations, advocacy documents, protocols, agreements, advocacy papers, laws
	Report	Document with the intention of relaying information to a specific audience for a specified purpose
	Review	Any collection or overview of research evidence
	Systematic review	Literature review focusing on a specific topic area, with stated methods including identification, appraisal, selection and synthesis of research evidence
		Includes databases searched, items searched, results of the search

## Results

Our sampling yielded 12 reports published by WHO, WB, and OECD between 2002 and 2013. Of the reports, two were categorized as health (H1, H2) [27,33], six were mixed (M1–M6) [25,34-38], and four were transport (T1–T4) [39-42] (Table 2). All publications were featured on the primary organization’s website and were available for download free of charge.

**Table 2:** **Selected publications on road traffic injury prevention and authorship analysis**

**Sector**	**Code**	**Year [Ref]**	**Title**	**Pages**	**Publishing groups**	**Number of authors**	**Health sector authors**	**Mixed sector authors**	**Transport sector authors**
Health	H1	2013 [27]	Global status report on road safety: Supporting a decade of action	303	WHO	N/A	N/A	N/A	N/A
Health	H2	2013 [33]	Strengthening road safety legislation: A practice and resource manual for countries	88	WHO	N/A	N/A	N/A	N/A
Mixed	M1	2009 [34]	Pedestrian Safety: A road safety manual for decision-makers and practitioners	114	WHO, WB, FIA	10	6	0	4
Mixed	M2	2009 [35]	Seatbelt Use: A road safety manual for decision-makers and practitioners	200	WHO, WB, FIA	10	6	1	3
Mixed	M3	2009 [36]	Speed Management: A road safety manual for decision-makers and practitioners	164	WHO, WB, FIA	5	0	0	5
Mixed	M4	2007 [37]	Drinking and Driving: A road safety manual for decision-makers and practitioners	149	WHO, WB, FIA	7	0	1	6
Mixed	M5	2004 [25]	World report on road traffic injury prevention	217	WHO, WB	7	5	1	1
Mixed	M6	2006 [38]	Helmets: A road safety manual for decision-makers and practitioners	147	WHO, WB, FIA	N/A	N/A	N/A	N/A
Transport	T1	2013 [39]	Guidelines for Mainstreaming Road Safety in Regional Trade Road Corridors	115	WB, EC, UN, ADB	2	0	1	1
Transport	T2	2008 [40]	Towards Zero: Ambitious road safety targets and the safe systems approach	241	OECD, ITF	10	0	2	8
Transport	T3	2002 [41]	Safety on roads, what’s the vision?	123	OECD	N/A*	N/A*	N/A*	N/A*
Transport	T4	2009 [42]	Country guidelines for the conduct of road safety management capacity reviews and the specification of lead agency reforms, investment strategies and safe system projects: implementing the recommendations of the world report on road traffic injury prevention	307	WB	2	0	1	1

ADB, African Development Bank; EC, European Commission; FIA Foundation, Federation Internationale de l’Automobile; ITF, International Transport Forum; OECD, Organization for Economic Co-operation and Development; UN, United Nations; WB, World Bank; WHO, World Health Organization.

*Study undertaken by road safety experts from OECD countries. Sectoral affiliation for 23 of 27 experts could be identified: 22 transport, 1 mixed.

N/A: Only an institutional author was provided.

### Publications and author networks

Author affiliations on health and transport sector reports were strongly aligned with the sector from which the report was published. In health sector reports, main authors were unnamed, but named contributors had primary affiliations in the public health sector (e.g., WHO, schools of public health). In transport sector reports, the majority of authors had primary affiliations in the transport sector (e.g., government affiliated transport departments, academic transport research centers). For the mixed sector reports, there was a clear majority grouping of authors from either within the health or transport sector, depending on the report subject topic. For example, report M1 and M2 (addressing pedestrian safety and seatbelt use, respectively) had a clear majority of authors originating from the health sector while M3 and M4 (addressing speed management and drinking and driving) had a clear majority of authors originating from the transport sector. M5 covered all of the aforementioned topics, but had a majority of authors from the health sector (Table 2).

### Interventions and evidence cited

Each of the reports summarized research evidence on two or more of the following intervention areas: alcohol use, road design, seatbelts/child restraints, speed limits, speeding, and vehicle design (Table 3). We found that regardless of which sector the reports originated from, the types of interventions covered overlapped considerably. There was some variation among the mixed reports, since they were topic-specific guides, each addressing a particular prominent risk factor for road traffic injuries. One mixed report (M5) [25], and one transport report (T4) [42] covered all seven intervention areas, while others used research evidence to summarize knowledge on two to six of the intervention areas.

**Table 3:** **Distribution of interventions covered across reports**

	**Report code**											
**Intervention**	**H1**	**H2**	M1*	M2*	M3*	M4*	**M5**	M6*	T1*	**T2**	**T3**	**T4**
Alcohol use	x	x	x			x	x			x	x	x
Road design	x		x		x		x		x	x		x
Seatbelts and restraints	x	x		x	x		x			x		x
Speed limits	x	x	x		x		x			x	x	x
Vehicle design	x			x	x	x	x		x	x	x	x
Speeding		x			x		x			x	x	x
Helmet use	x	x					x	x			x	x

*Reports M1, M2, M3, M4, M6, and T1 are topic specific reports for policymakers, so each report may have covered fewer topics than other reports.

From the 12 selected reports, a total of 419 citations from the report sections covering our 7 intervention groups were extracted. We categorized the type of research evidence (e.g., report, systematic review, observational study) and its organizational affiliation (e.g., government agency, peer-reviewed journal) for each citation. A summary on the proportion of evidence cited by sector can be found in Table 4.

**Table 4:** **Types of research evidence cited and institutional affiliations of cited research evidence**

		**Organizational affiliation**					
		**Health**	**%**	**Mixed**	**%**	**Transport**	**%**
**Evidence type**	Report	22	32.35%	86	31.85%	56	69.14%
	Observational study	18	26.47%	53	19.63%	4	4.94%
	Review	8	11.76%	26	9.63%	8	9.88%
	Guidelines	4	5.88%	20	7.41%	2	2.47%
	Other	8	11.76%	22	8.15%	3	3.70%
	Case study	0	0.00%	19	7.04%	1	1.23%
	Systematic review	5	7.35%	17	6.30%	0	0.00%
	Experimental	0	0.00%	5	1.85%	1	1.23%
	Modeling	0	0.00%	8	2.96%	5	6.17%
	Mechanical simulations	2	2.94%	5	1.85%	0	0.00%
	Economic analysis	1	1.47%	6	2.22%	1	1.23%
	Qualitative	0	0.00%	3	1.11%	0	0.00%
	Total	68	100%	270	100%	81	100%
**Institutional affiliation**	Government agency	9	13.24%	84	31.11%	36	44.44%
	Peer-reviewed journal	27	39.71%	73	27.04%	4	4.94%
	Multilateral government organization	13	19.12%	36	13.33%	18	22.22%
	Non-governmental organization	3	4.41%	29	10.74%	8	9.88%
	Academia	2	2.94%	7	2.59%	5	6.17%
	Private	0	0.00%	4	1.48%	4	4.94%
	Conference	4	5.88%	18	6.67%	3	3.70%
	Other	10	14.71%	19	7.04%	3	3.70%
	Total	68	100%	270	100%	81	100%

There was a stark difference in the types of prevailing evidence cited across the health and transport reports. Evidence in the health sector was split amongst reports (32.4%), observational studies (26.5%), and systematic and non-systematic reviews (19.1%), while evidence cited from the transport sector was dominated by agency and organizational reports (69.1%). Approximately 7.4% of citations from the health sector were systematic reviews, while the transport sector cited none.

Institutional affiliations of evidence cited also varied considerably by the sector from which the report was published. The transport sector had the highest proportion of government agencies (44.4%) and multilateral organization (22.2%) reports, while the health sector had the highest proportion of evidence from peer-reviewed academic journals (39.7%). Though government agencies were strongly present in all sector reports, the striking difference arises in the presence of peer-reviewed literature in health, but not the transport sector.

### Report recommendations

By and large, despite the differing make-up of evidence types that informed reports across the health and transport sectors, recommendations and conclusions across the reports were similar. The conclusions of all reports were non-specific and focused on the process of implementing RTIP policies and programs, rather than recommending specific interventions. The conclusions generally stated that policymakers should first examine their own country/district’s situation through suggested activities and processes (e.g., assess current problems, set realistic targets, assess implementation capacity, identify lead agencies, etc.). Only after assessing their own situation, should they utilize the evidence on interventions included in the reports, match the evidence to their own situation, and implement accordingly.

### Indicators

Seven indicators were tracked across the 12 reports (summarized in Table 5). We found that three indicators (number of road traffic injuries, number of deaths from road traffic injuries, and losses in gross national product attributed to road traffic injuries) were used consistently across almost all the selected reports as a framing tool to describe the magnitude of the road traffic injuries across countries. The fatalities per 100,000 population indicator was also present in all reports but one, though data were not explicitly available for four. The fatalities per 10,000 vehicles, fatalities per vehicle kilometer traveled, and disability-adjusted life years (DALYs) indicators were seldom used across reports originating from any sector, despite being deemed a common indicator for RTIP [25]. DALYs were only used in two mixed reports, perhaps suggesting that it could be a difficult summary measure to understand for non-health sector audiences*.* Despite the reports being published from organizations rooted in different sectors, with differing focus in the types of evidence cited, we saw no substantive difference in the use of indicators across sectors.

**Table 5:** **Use of indicators across reports**

**Indicator**	**Definition**	**H1**	**H2**	**M1**	**M2**	**M3**	**M4**	**M5**	**M6**	**T1**	**T2**	**T3**	**T4**
Number of injuries	Absolute figure indicating the number of people injured in road traffic crashes; injuries sustained may be serious or slight	x	x	x	x	x	x	x	x	x	x	x	x
Number of deaths	Absolute figure indicating the number of people who die as a result of a road traffic crash	x	x	x	x	x	x	x	x	x	x	x	x
Fatalities per 10,000 vehicles	Relative figure showing ratio of fatalities to motor vehicles					*		x					x
Fatalities per 100,000 population	Relative figure showing ratio of fatalities to population	*		*	*	*	x	x	x	x	x	x	x
Fatalities per vehicle kilometer traveled	Number of road deaths per kilometer traveled					*	*	x			x	*	*
Disability-adjusted life year (DALY)	Measures healthy life years lost due to disability and mortality							x	x				
Losses as fraction of gross national product (GNP)	Percent of GNP losses attributed to crashes	x		x	x	x	x	x		x	x	x	x

*Indicator was described in the report but data were not reported; x, Indicator was explicitly used and reported in the report.

## Discussion

This analysis used selected policy documents on RTIP to explore differences in the nature of evidence used in reports originating from different sectors. In summary, our findings comprise the following points. First, there was clear representation of distinct authorship networks in each sector, with the authors predominantly belonging to the sector from which the document was published. Second, the types of evidence cited in each report were different across sectors, with health sector reports emphasizing observational studies and reviews (both systematic and non-systematic) in peer-reviewed journals, while transport sector reports drew on government agency reports. Third, despite substantive differences in the types of evidence cited, concluding recommendations of these reports were similar, focusing on generic policy processes rather than specific interventions. Fourth, indicators were used consistently across reports from different sectors. We reflect on the nature of our findings, limitations in study design, and the implications of the findings for future action and further research.

The clustered nature of author networks on transport and health reports aligns with Haas’ concept of epistemic communities, whereby networks of professionals with recognized expertise share i) causal beliefs which serve as the basis for possible policy actions and desired outcomes and ii) notions of validity for weighing and validating information within the domain of expertise [43]. Reports from the health and transport sector each preferentially cited different types of evidence, suggesting that different forms of evidence were favored by different sectors; this, in turn, may reflect differing notions of knowledge validity held by each sector. It is often thought that without a coherent, and shared, framing of a policy problem, both internally and externally [44], it will be difficult for a policy issue to gain prominence. However, RTIP has recently been quite successful in achieving policy prominence, and appears to have coalesced around somewhat common problem statements and solutions, despite significant differences in epistemologies across the two component sectors. As others have suggested, epistemic communities may not need to be monolithic to be successful so long as they can achieve consensus around the need for reform, and a road map for reform [45]. The relatively vague nature of the recommendations identified in the study likely reflects the fact that these were international reports that needed to be relevant to countries with diverse contexts. There is also a possibility that difference in perspectives between the different sectoral epistemic communities prevented the formulation of more concrete recommendations, but this would require additional investigation.

The differing valuations of evidence across the two communities may stem from their respective field’s historical development. The emphasis in the health community on observational studies and systematic reviews matches with common valuations of evidence within the biomedical/epidemiological tradition [46], and indeed public health developed out of the biomedical research paradigm, with a heavy emphasis on ‘evidence-based’ approaches utilizing peer-reviewed experimental and observational study designs [47]. Transport safety, by contrast, evolved into a distinct entity from the purview of police departments to engineers [48]. During the past few decades, OECD countries have managed to achieve significant improvements in road safety through a ‘safe systems’ approach that reflects complexity science and systems engineering. This approach is firmly rooted in a pragmatic perspective addressing the reliability of the overall system, and logistics for maintaining it, in practice seeking to layer multiple interconnected protections relating to road infrastructure, vehicle design, and road-user awareness [49]. These legacies shaped each sector’s early development and continue to contribute to the dominant knowledge paradigms in each. Notably, the transport sector draws heavily upon government reports that reflect practitioner experience of implementing different systems, whereas the health sector draws more upon research in peer-reviewed journals. However, we acknowledge that RTIP is increasingly investigated universally via the host-agent-environment, which includes engineering and social/behavioral change science frames.

Recent literature has heavily emphasized the importance of interdisciplinarity and transdisciplinarity in public health research [50,51]. The question that we have explored herein, around the intersectoral application of evidence, is distinct from, but related to, that of interdisciplinarity. While our study has focused on the nature of evidence applied to policy and decision-making in different sectors, inter- and transdisciplinarity concern the prior step of knowledge creation and the role of different disciplinary methods and perspectives in this process. Despite this distinction, the literature on interdisciplinarity and transdisciplinarity is of relevance to the question posed herein. First, it is relevant in a very direct way. While there are dominant disciplines in each sector, researchers working within separate health sector and transport sector evidentiary paradigms already appear to draw upon a mix of disciplinary perspectives. It seems possible that the more interdisciplinary each sector’s work becomes, the more accessible it may be to the other sector. Second, there are likely to be relevant lessons from efforts to promote greater interdisciplinarity that concern, for example, strategies to promote greater understanding and trust across different disciplines, mechanisms to promote inter-agency collaboration [51], and the importance of cross-boundary spanners (see below).

### Study limitations

This was an exploratory study designed to investigate an issue on which there has been very little prior research. The study has a number of limitations. First, sampling of reports from each of the sectors was based on expert opinion and potential for replicability is accordingly limited. While we originally intended to use citation counts as a measure of prominence to select reports, limitations in the scope of grey literature citation tracking prevented this and hence expert consultation was used instead. Second, our study targeted global reports, and perhaps as a consequence of this and the great heterogeneity in country contexts across the world, the recommendations presented in each report were quite generic. A similar analysis at national or sub-national level may have identified more specific policy recommendations. This is important as it is impossible to conclude whether the non-specific recommendations are more reflective of country heterogeneity or of lack of agreement on strategies across transport and health sector actors. Third, our study considers only one intersectoral topic area; it is possible that findings may differ considerably for other topics and other sectors where evidentiary paradigms differ. Finally, while we have classified people according to their current institutional affiliation, it is possible that some researchers trained in one paradigm (e.g., health) may have crossed over to work in the other sector. We do not have empirical data on how common this is, but based on both our own experience and a scan of the online CVs of authors of the reports studied in this paper it appears relatively rare.

### Implications

Given the exploratory nature of this study, it would be inappropriate to draw hard and fast conclusions about the implications of this work; nonetheless, a number of points are worth reflecting on.

First, the author analysis helped identify individuals (such as the one transport and one mixed sector author on the WHO report [25]) who form critical bridges across the two sectors. Such individuals, sometimes known as ‘boundary spanners’ or ‘brokers’, can facilitate transactions and the flow of information among disparate groups, such as those sharing different notions of evidence, enabling communication and better facilitating intersectoral collaboration [52]. It is important to recognize these individuals, the skills they possess, the privileged positions they occupy within professional networks, and the potential that they have in facilitating intersectoral action and, thus, to nurture their development.

With increasing emphasis on research dissemination to bridge the evidence-to-practice gap, researchers are engaging in ‘push’ activities to identify actionable messages from their research, tailor them to specific audiences, and work with groups to deliver them [53]. Our findings on the varying notions of knowledge validity amongst the transport and health sectors further emphasize the importance of tailoring messages, notably for policymakers involved in intersectoral issues who are unfamiliar with evidence from other sectors. Researchers should be cognizant that, especially on intersectoral problems, their audience expands beyond their home sector, and researchers and decision-makers from other sectors may benefit from understanding the value of the research and its methods. Additionally, researchers should disseminate findings through channels that have high knowledge validity not only in their own sector, but are also targeted towards other sectors.

Finally, while we started this study with some notions of the likely differences in evidence between the health and transport sectors regarding RTIP, the analysis forced us to think more deeply and reach a refined understanding of the historical evolution of the transport sector and the different disciplinary perspectives that inform thinking within the sector. In turn, this has led to a clearer understanding of the likely points of tension between the two sectors in terms of which types of evidence are valued. It seems likely that similar analyses for other intersectoral issues may be valuable in promoting improved mutual understanding across sectors.

### Future research

This study focused on one intersectoral issue (RTIP) and two primary fields (public health and transport) that have informed the development of this policy area. As noted in the section on study weaknesses, findings may vary substantially in other intersectoral fields and the global nature of policy reports reviewed likely contributed to the relatively generic policy recommendations found in both sectors, despite apparently substantive differences in use of evidence. In light of this, we would encourage others to undertake similar reviews for other intersectoral policy issues, as well as to extend analyses of this nature to the national level. It would be particularly interesting to contrast findings for highly contested intersectoral policy issues with less contested ones.

There are many further questions about the process of evidence use around intersectoral policies that could shed light on how significant a barrier evidentiary traditions are to collaboration and good practices supporting evidence use across sectors. For example, qualitative studies could explore the role of research evidence in the intersectoral policy process: are both sectors equally responsible for providing the evidentiary basis for policy decisions and what determines this? How is research evidence valued from one sector to another when it does not meet their community’s notion of knowledge validity? Further, social network analysis could cast light on the constituent communities within each sector, how they relate, and which individuals are well situated to play a broker role between communities. Finally, further research could be conducted to investigate the nature of recommendations among other intersectoral issues where there is less fragmentation of the policy community.

## Conclusions

Intersectoral action for health has tremendous potential but little research has been done on how research evidence is used when sectors from different scientific paradigms come to work together. Through documentary analysis of reports originating in the health and transport sectors, we demonstrated that, for road traffic injury prevention, the health and transport communities exist in separate scientific paradigms and within their own, albeit connected, epistemic communities. The health and transport sectors valued and drew on different types of evidence to support their recommended interventions. For road traffic injury prevention, the two communities have managed to create a sufficiently coherent problem statement and vision for the issue to have received a relatively high level global recognition. However, translating this commitment to address the problem into practical strategies for intersectoral action may require even greater collaboration, and shared understanding of the relevance of evidence from both sectors.

## Abbreviations

ITF: International Transport Forum
RTIP: Road traffic injury prevention
OECD: Organization for Economic Cooperation and Development
UN: United Nations
WB: World Bank
WHO: World Health Organization
